# The impact of exposure to tobacco smoking and maternal trauma in fetal life on risk of migraine

**DOI:** 10.3389/fnins.2023.1191091

**Published:** 2023-06-29

**Authors:** Magdalena Kobus, Aneta Sitek, Bogusław Antoszewski, Jacek J. Rożniecki, Jacek Pełka, Elżbieta Żądzińska

**Affiliations:** ^1^Department of Anthropology, Faculty of Biology and Environmental Protection, University of Lodz, Łódź, Poland; ^2^Department of Plastic, Reconstructive and Esthetic Surgery, Institute of Surgery, Medical University of Lodz, Łódź, Poland; ^3^Department of Neurology, Stroke and Neurorehabilitation, Medical University of Lodz, Lodz, Poland; ^4^Department of Neurology, Norbert Barlicki Memory University Teaching Hospital, Lodz, Poland; ^5^Biological Anthropology and Comparative Anatomy Research Unit, School of Medicine, The University of Adelaide, Adelaide, SA, Australia

**Keywords:** migraine, headache, pregnancy, tobacco smoking, trauma, stress, prenatal period

## Abstract

**Introduction:**

Prenatal period is the key time in human development. Many prenatal factors are well-known and increase the risk of developing diseases’ after birth. Few studies indicated the link between the prenatal period and the prevalence of migraine in childhood and adolescence so far. We decided to broaden current knowledge and investigate whether the prenatal factors influence the prevalence of migraine in adulthood. The objective of this study is to provide evidence of relationship between *in utero* environment and risk of migraine.

**Methods:**

In total 266 females (136 in the migraine group, 130 in the control group) and 80 males (35 in the migraine group, 45 in the control group), aged 18–65 participated in the study. The quality of prenatal environment was characterized on the basis of mother’s and father’s education, tobacco smoke exposure, alcohol consumption, and traumatic event during pregnancy, which are considered as prenatal factors and affect on fetal development.

**Results:**

Migraine occurrence in adulthood was significantly associated with maternal tobacco smoking during pregnancy (OR 3.42, 95% CI 1.54–7.61, *p* = 0.036) and traumatic event during pregnancy (OR 2.27, 95% CI 1.24–4.13, *p* = 0.020).

**Discussion:**

Our study suggests that the fetal programming effect of tobacco smoking exposure and maternal trauma is not limited to prenatal life and is suggested as having a role in adulthood. Our findings support evidence that migraine adulthood can be partly influenced by early life conditions.

## 1. Introduction

Primary headaches are the most prevalent non-communicable diseases (NCDs), but its public health impact have been neglected. In Europe, almost 80% of the adult population (aged 18–65) have suffered from tension-type headache (WHO, 2011). Migraine headache is less prevalent, unrelated to any other medical condition and ranked among the 10 leading causes of disabilities worldwide (GBD 2016 Headache Collaborators, 2018). Migraine is more than a moderate to severe headache on one side of a head. Furthermore, headache is often accompanied by nausea, vomiting, light and sound sensitivity, and visual, sensory, speech, or motor symptoms (IHS, 2018). Migraine background involves genetic and environmental factors, but little research has been done about prenatal factors (Ulrich et al., 1999; Mulder et al., 2003; Sutherland and Griffiths, 2017; Kobus et al., 2021).

This type of primary headache affects 14% people (aged from 0 to over 65 years) and its prevalence is significantly related to the sex (Stovner et al., 2022). In general population migraine is more common in women than in men (female-to-male ratio is 3:1) (Goadsby et al., 2002; Dodick, 2006). In childhood and adolescence the prevalence ranges from 7.7 to 9.1% (Abu-Arafeh et al., 2010; Wober-Bingol, 2013). If migraine onsets appeared in childhood, about half of the people will also suffer from it in adulthood (Dilling-Ostrowska, 2005). The risk is higher when migraine started manifesting itself during puberty (Barnes, 2015). In the study, we considered prevalence of migraine in adults as the delayed consequence of disturbances in the prenatal period of life. NCDs have common risk factors, which are related to the lifestyle. However, crucial role lies early in life conditions, the period from conception. The Developmental Origins of Health and Disease (DOHaD) theory describes long-term risk of the major NCDs (Gluckman et al., 2010).

The concept of intrauterine programming is a part of the DOHaD theory (Barker, 1990). For example, Barker’s hypothesis used birthweight as an indicator of the developmental processes with the long-term effects (Kwon and Kim, 2017). In general, pregnancy is a period of particular vulnerability to diseases that affects both mother’s and fetus’ health. Life challenging events (e.g., trauma; Zadzinska et al., 2013; Hjort et al., 2021), drugs (e.g., alcohol or tobacco exposure; Committee Opinion No 721, 2017; Popova et al., 2019) and care of both parents based on education are key factors of early human development (Mangrio et al., 2011).

The prenatal period is crucial in human life and is also the time of preparation for taking on new roles—mother and father. Parental behavior affects fetal and child development. So far it is known that tobacco exposure and alcohol consumption during pregnancy have been risk factors of migraine in offspring (Arruda et al., 2011; Bigal and Lipton, 2011; Fabbri et al., 2012). Most of these studies have been related to the prevalence of migraines in childhood or adolescence. Episodic, frequent, and chronic headaches are associated with sleep disorders (Bruni et al., 1997), peer problems (Strine et al., 2006), depression (Gazerani, 2021), anxiety (Maratos and Wilkinson, 1982; Gazerani, 2021), and high suicidal risk (Wang et al., 2007) in the aforementioned group.

There is little known about prenatal factors associated with increased migraine risk in adulthood. The main aim of this study was to examine if parental education and behavior during pregnancy are associated with development of migraine in adults. To the best of our knowledge, this is the first study that considered association of early exposure to selected prenatal factors in adult migraineurs.

## 2. Materials and methods

### 2.1. Participants

The study was conducted according to the guidelines of the Declaration of Helsinki and approved by the Ethical Commission at the University of Lodz (no. 16/KBBN-UŁ/III/2018). Written informed consent form was obtained from all participants after receiving detailed study information. Patients were recruited in Norbert Barlicki Memorial University Teaching Hospital No. 1 in Lodz. The study included two groups: the group of 171 adults from neurological clinic, aged 18–65 (35 males, 136 females) and the control group of 175 adults from plastic surgery clinic, aged 20–65 (45 males, 130 females). We excluded 23 individuals over 65 owing to their advanced age (66–76-year-old), which implied a significant loss of memory and a limitation in terms of information about the their parents behavior during pregnancy (Lezak et al., 2012; Murman, 2015). Participants were examined by neurologist and categorized to the migraine group or the healthy individuals group without neurological diseases (according to the International Classification of Headache Disorders, third edition; IHS, 2018). Patients with chronic diseases were excluded from the study during the preliminary interview.

### 2.2. Questionnaire

We gathered data from the questionnaire, which covered the information about the participants’ prenatal period and was declared by them. Beside sex and age of respondents, the questionnaire included parental- and prenatal-related questions about: mother’s and father’s education, tobacco smoke exposure, alcohol consumption and traumatic event during pregnancy. In addition, we controlled hereditary trait and included a question about family history of migraine. Parental education levels were categorized into three sections: primary or vocational education, secondary education or Bachelor’s course graduates, and Master’s course graduates. Exposure to tobacco smoke during pregnancy was divided into maternal smoking (MSDP) and maternal passive smoking (MPSDP), defined as smoking by close relatives of the pregnant woman. Maternal trauma during pregnancy was related to the major stressful life events, which were given as the examples (death of a spouse or close person, divorce, personal injury or illness etc.; Holmes and Rahe, 1967). Answers of the prenatal-related questions were categorized as “yes”/“no”/“do not know,” family history of migraine as “yes”/“no.” In the both study groups, there were no missing data for each variable of interest. Research in adult groups may be limited with frequency of “do not know” reply than in pediatric groups, when parents complete questionnaires themselves.

### 2.3. Statistical analysis

Statistical analysis of the results was performed in the STATISTICA 13.0 program. All statistical calculations were carried out in spreadsheets after anonymization of the data. At the initial stage, we verified the accuracy of the questionnaire responses by using Test–Retest Reliability (Bland and Altman, 1986). Student *t*-test was used to access age differences; chi^2^ test was used to access sex and parental variables’ differences. We used stepwise logistic regression model for odds ratios (ORs) calculation with 95% confidence interval (CI).

## 3. Results

Median age for females was 39 (±12.6) years in migraine group and 40.5 (±11.3) years in control group. Median age for males was 36 (±13.0) years in migraine group and 34 (±10.6) years in control group. No statistically significant differences were found in age between migraine and control group (*p* = 0.384) and sex (*p* = 0.247) in both study groups. We repeated the questionnaire 1 month after the date of the first data collection on 30 participants (migraine group: *N* = 15, control group: *N* = 15). The results of the repeated questionnaires showed 100% agreement with the previous questionnaires. Only in the case of the question concerning alcohol consumption, the agreement was slightly lower (93%), which indicates a high reliability of the data obtained through the questionnaires.

Table 1 presents distribution of variables that were taken into consideration in the study. In the case of parents’ education, no significant differences were observed in either mother’s (*p* = 0.553) or father’s (*p* = 0.192) education level. Significant differences were found in terms of maternal traumatic event (*p* = 0.022), MSDP (*p* = 0.003), but not MPSDP (*p* = 0.095). The vast majority of respondents in the both study groups denied that their mother drunk alcohol during pregnancy (*p* = 0.282). In case of heritable trait, significant differences in family history of migraine were observed (*p* < 0.001).

**Table 1:** Distribution of parental and prenatal factors.

Factor	Answer	Migraine group (F = 136, M = 35)	Control group (F = 130, M = 45)	Chi^2^	*p*
		N (%)	N (%)		
Mother’s education level	Master’s course graduates	39 (22.8%)	46 (26.3%)	1.19	0.553
	Secondary education or Bachelor’s course graduates	55 (32.2%)	60 (34.3%)		
	Primary or vocational education	77 (45%)	69 (39.4%)		
Father’s education level	Master’s course graduates	45 (26.3%)	37 (21.1%)	3.30	0.192
	Secondary education or Bachelor’s course graduates	44 (25.7%)	60 (34.3%)		
	Primary or vocational education	82 (48%)	78 (44.6%)		
Maternal smoking (MSDP)	Yes	26 (15.2%)	9 (5.1%)	11.52	0.003
	No	129 (75.4%)	155 (88.6%)		
	Do not know	16 (9.4%)	11 (6.3%)		
Maternal passive smoking (MPSDP)	Yes	91 (55.2%)	75 (42.9%)	4.70	0.095
	No	72 (42.1%)	94 (53.7%)		
	Do not know	8 (4.7%)	6 (3.4%)		
Alcohol consumption	Yes	8 (4.7%)	3 (1.7%)	2.53	0.282
	No	150 (87.7%)	157 (89.7%)		
	Do not know	13 (7.6%)	15 (8.6%)		
Traumatic event	Yes	39 (22.8%)	21 (12%)	7.66	0.022
	No	104 (60.8%)	127 (72.6%)		
	Do not know	28 (16.4%)	27 (15.4%)		
Family history of migraine	Yes	101 (59.1%)	13 (7.4%)	104.4	< 0.001
	No	70 (40.9%)	162 (92.6%)		

The standard logistic regression model is presented in Table 2 and the final model in Table 3. The stepwise logistic regression model showed that among the parental and prenatal factors MSDP and traumatic event during pregnancy significantly elevated risk of migraine in offspring (Table 3). MSDP increased the risk of migraine almost 4-fold (OR 3.42, 95% CI 1.54–7.61, *p* = 0.036), while maternal trauma increased the risk more than 2-fold (OR 2.27, 95% CI 1.24–4.13, *p* = 0.020). The effects of aforementioned factors explained 7% of migraine prevalence in Polish adults (Nagelkerke *R*^2^ = 0.07).

**Table 2:** Standard model of logistic regression presenting the probability of migraine prevalence in adults.

Effect	Nagelkerke *R*^2^ = 0.11					
	Coefficient	Standard error	Wald value	OR	95% CI for OR	*p*
Mother’s education level: Master’s course graduates^1^	−0.210	0.210	0.999	0.64	0.30–1.37	0.317
Mother’s education level: Secondary education or Bachelor’s course graduates^1^	−0.024	0.175	0.018	0.77	0.40–1.48	0.892
Father’s education level: Master’s course graduates^1^	0.361	0.213	2.867	1.60	0.75–3.42	0.090
Father’s education level: Secondary education or Bachelor’s course graduates^1^	−0.251	0.180	1.948	0.87	0.45–1.67	0.163
Maternal smoking (MSDP): yes^2^	0.632	0.313	4.091	3.62	1.52–8.64	0.043
Maternal smoking (MSDP): do not know^2^	0.023	0.320	0.005	1.97	0.80–4.84	0.943
Maternal passive smoking (MPSDP): yes^2^	0.039	0.221	0.032	1.44	0.91–2.27	0.859
Maternal passive smoking (MPSDP): do not know^2^	0.286	0.381	0.564	1.84	0.59–5.80	0.453
Alcohol consumption: yes^2^	0.555	0.507	1.199	1.78	0.41–7.74	0.274
Alcohol consumption: do not know^2^	−0.533	0.369	2.084	0.60	0.25–1.44	0.149
Traumatic event: yes^2^	0.473	0.216	4.803	2.12	1.15–3.90	0.028
Traumatic event: do not know^2^	−0.196	0.219	0.797	1.09	0.58–2.03	0.372

1 vs primary or vocational education.

2 vs no.

OR, odds ratio; p, probability; Wald test was used to check regression variables’ relevance.

**Table 3:** Stepwise logistic regression model presenting the probability of migraine prevalence in adults.

Effect	Nagelkerke *R*^2^ = 0.07					
	Coefficient	Standard error	Wald value	OR	95% CI for OR	*p*
Maternal smoking (MSDP): yes^1^	0.618	0.295	4.385	3.42	1.54–7.61	0.036
Maternal smoking (MSDP): do not know^1^	−0.007	0.301	0.001	1.83	0.80–4.17	0.981
Traumatic event: yes^1^	0.492	0.211	5.407	2.27	1.24–4.13	0.020
Traumatic event: do not know^1^	−0.164	0.214	0.590	1.18	0.64–2.17	0.442

1 vs no.

OR, odds ratio; p, probability; Wald test was used to check regression variables’ relevance.

## 4. Discussion

During an individual development there are critical windows, such as fetal period, when fetus reacts to intrauterine environment and adapts to its conditions. During prenatal development, any distrubing conditions may bring delayed consequences during ontogenesis (Barker, 1990; Zadzinska et al., 2016; Koziel et al., 2018). As a result, possible adulthood diseases are being “programmed”—this process is persistent, but further determined by gene expression (Warner and Ozanne, 2010; Rinaudo and Wang, 2012). Intrauterine programming is based on genetic determinants and adaptive abilities of fetus to environmental conditions.

One of the possible effects of disturbances in prenatal development is inadequate birth weight, according to the sex and gestational age. Abnormal birth weight and its influence on risk of migraine was investigated by Borte et al. (2017). Researchers showed that growth restriction *in utero* was related to the greater risk of migraine in adults (Borte et al., 2017). In addition to the birth phenotype, the latest research (2022) showed for the first time that migraine was associated with physical performance results (Kobus et al., 2022a) and skin phenotype (Kobus et al., 2022b). It is intriguing, because at the prenatal stage melanocytes and nervous system share common origin.

In this study, among analyzed variables, maternal smoking during pregnancy (MSDP) had an effect on the development of migraine in adulthood. MSDP causes numerous health problems to the fetus including preterm birth (Kondracki and Hofferth, 2019), admission to the neonatal intensive care unit (Nichols et al., 2019), and e.g., has harmful influence on head growth and brain development (Scherman et al., 2018). The disturbances in head growth can be assessed by simple anthropometric measurement like head circumference (HC) immediately after birth, which is taken as an alternative indicator of brain size assessment (Bartholomeusz et al., 2002). Clinicians agree that HC measurement is adequate method identifying disturbances in brain growth patterns (Sacco et al., 2015). HC was significantly smaller for gestational age compared to offspring of non-smoking mothers (Kallen, 2000; Fenercioglu et al., 2009; Shiohama et al., 2021). Swedish cohort study (*N* = 1,362,169) showed that MSDP increased risk of reduced HC in accordance with daily cigarettes consumption from 1.5-fold (<10 cigarettes per day, OR 1.48) to 1.7-fold (>10 cigarettes per day, OR 1.74; Kallen, 2000). Another cohort study (*N* = 84,856), conducted in Japan, showed that MSDP resulted in 1.7-fold (1–5 cigarettes per day, OR 1.69) to over 5-fold (5–10 cigarettes per day, OR 5.19) higher risk of smaller HC than in infants unexposed to tobacco (Shiohama et al., 2021). MSDP affects head shape and size beyond fetal period. Polish study showed negative influence on postnatal head parameters in boys group, but did not in girls group (Koziel et al., 2018). As a matter of fact, males are more sensitive to environmental conditions *in utero* (Zadzinska and Rosset, 2013; Sutherland and Brunwasser, 2018). Additionally, intrauterine exposure to maternal smoking is related to reduce volume of cortical gray matter in childhood (El Marroun et al., 2014; Chatterton et al., 2017). Message about impaired prenatal and postnatal growth of offspring exposed to MSDP (Fenercioglu et al., 2009) and long-term consequences across ontogenesis have to be clearly emphasized (Richmond et al., 2015).

Fetal brain growth is dependent on oxygen supply. The major component in tobacco smoke is nicotine, which crosses placenta, enters fetal circulation and leads to contraction of placenta‘s blood vessels. Next ingredient of smoke is carbon monoxide, which has an ability to bind with hemoglobin. As a result occurs a limitation of the oxygen supply to the fetus. Protective mechanism reduces blood flow to placenta to limit adverse neuronal effect, which results in smaller transcerebellar diameter and lower brain volume (Roza et al., 2007). Reduced process of oxygen delivery results in lower birthweight, decreased placental weight (Wang et al., 2014) and increased risk of miscarriage (Mishra et al., 2000). In the recent study, Pietersma et al. (2022) examined for the first time relationship between MSDP before and after conception (14 vs. 10 weeks) and embryonic development. Virtual Reality techniques were used to access development and morphology of embryo. MSDP was associated with delayed embryonic morphological development, decreased fetal size, and lower birthweight. Embryos which were exposed to tobacco were not be able to “catch-up” during pregnancy as the result they were born with lower birthweight than expected for sex and gestational age. The major key finding of this study was that the delayed in embryonic development caused by MSDP during periconception period was associated with lower fetal growth. Prenatal ultrasound measurements were conducted at around 20 weeks of pregnancy. Researchers showed the importance of smoking cessation before conception. Taking all into consideration, the findings of this study emphasized that the best recommendation is smoking cessation at point of planning to become pregnant. Tobacco exposure affects embryo from the earliest stages of pregnancy. This underlines the importance of public health initiatives promoting education and preconception care (Pietersma et al., 2022).

Fabbri et al. (2012) reported in the group of children (8–10-year old) higher migraine prevalence in individuals with history of MSDP. In population-based study of a group of Brazilian children (5–12-year old), prenatal exposure to tobacco was associated with headache chronification (MSDP OR 2.29; MPSDP OR 4.2; Arruda et al., 2011). Our study showed that MSDP is one of the most important modifiable prenatal factor of migraine in the adult group (OR 3.42). This result emphasized the importance of education intervention programs prior to pregnancy. MSDP is serious a problem from public health perspective in Poland in the light of the fact that smoking is the most common addiction of reproductive aged women (Olejniczak et al., 2021). Although MPSDP was not significant in our study, it is noteworthy that smoking by close relatives of the pregnant woman was very common in both groups (migraine group: 55.2%, control group: 42.9%).

Parental smoking during pregnancy involves both parents (MSDP/MPSDP). This behavior increases risk of complications during pregnancy and put offspring at risk for certain birth defects before they are born. Environmental exposure to tobacco smoke (referred to MPSDP) is associated with mother’s health problems during pregnancy such as gestational diabetes (Morales-Suarez-Varela et al., 2022). Dutch population-based study (*N* = 1,858) showed that father’s support is a key element in reducing MSDP (Scheffers-van Schayck et al., 2019). Mother and fetus benefit from father’s involvement during perinatal and postpartum support (Martin et al., 2007; Firouzan et al., 2018). Involved father has influence on positive and negative maternal health behaviors and reducing mother’s stress level owing to emotional and financial support (Yolanda and Padilla, 2001). Moreover, father’s attitude toward participation in antenatal and perinatal care is linked with higher education level and bigger income (Maken et al., 2018). Low socioeconomic status of family and low education level are associated with prevalence of primary headaches, in particular migraine (Bigal et al., 2007; Chu et al., 2013; Stewart et al., 2013).

In addition to the modifiable parental factors (behavior and daily habits), the mother and fetus may be exposed to unpredictable traumatic event during pregnancy. In the migraine group, a traumatic event during pregnancy occurred in almost one in four pregnancies (22.8%), compared with only one in 10 in the control group (12%). Individuals whose mother experienced traumatic stress during pregnancy had over 2-fold increased risk of migraine (OR 2.27) in adulthood. The examples given in the questionnaire were classified as the most stressful life events according to the Social Readjustment Rating Scale. For instance, death of a spouse is rated as 100 units (from 0 to 100; divorce: 73 units, personal injury or illness: 53 units; Holmes and Rahe, 1967). Some biological mechanisms are reported to explain how maternal traumatic stress affects offspring development. Elevated levels of fetal glucocorticoids may impair intrauterine blood flow and affect the neurodevelopment, significantly affects the development of the brain (Fitzgerald et al., 2021). Future research on a possible neurobiological mechanism linking early stress and later migraine occurrence is needed.

Parental stress is associated with higher frequency and intensity of migraine in school-age children (Anttila et al., 2004; Smirni and Carotenuto, 2021). This fact remarks on the need to expand the migraine educational programs to family members (Esposito et al., 2013). Dealing with family issues in clinical practice may reduce the incidence of stress-related migraine in offspring (Hammond et al., 2019).

The influence of family history of migraine on its onset risk has been well described in family and twin studies. Heritability is estimated at 30–60% (Sutherland et al., 2019). In our study rate of positive family history was high (59%). Aside from family history of migraine, sex and age are the most common risk factors. Migraine prevalence in general population is three times higher in women than in men (Goadsby et al., 2002). The migraine occurrence in adulthood is fluctuating and is the most common in middle-aged people. In fact, it is the most burdensome among young adults and middle-aged women (GBD 2016 Headache Collaborators, 2018). In our study, median age for female migraineurs was 39 years and for male migraineurs was 36 years. It showed that migraine often affects people during productive period. From public health perspective, it is important to maintain education about negative influence of tobacco smoking, vaping and passive smoking on fetus. Pregnant women and their close relatives should remember that there is no risk-free level of tobacco exposure and alcohol exposure as well (Lucchini et al., 2021). It is indicated that smoking and alcohol consumption by father during pregnancy were related to worse offspring health (Easey and Sharp, 2021). Both parents are responsible for their child since early human development stages.

## 5. Conclusion

Taken together, our study broadens the findings on prenatal factors of migraine. Maternal smoking and traumatic event during pregnancy seem to be significant prenatal factors of migraine in offspring and in adulthood. Migraine in adulthood can be perceived as the delayed consequence of maternal smoking and trauma. In this study, association of parental behavior and maternal trauma during pregnancy with migraine risk were examined for the first time among adults. Nonetheless, longitudinal studies about the influence of prenatal factors and migraine frequency during all stages of ontogenesis are still needed, since they have not been conducted so far.

## Data availability statement

The raw data supporting the conclusions of this article will be made available by the authors, without undue reservation.

## Ethics statement

The studies involving human participants were reviewed and approved by the Ethical Commission at the University of Lodz (no. 16/KBBN-UŁ/III/2018). The patients/participants provided their written informed consent to participate in this study.

## Author contributions

MK, AS, BA, JJR, and EŻ: conceptualization and critical comments. MK, AS, and JP: acquisition. MK and AS: analysis. MK, AS, and EŻ: interpretation of data. MK: draft version and final version. All authors contributed to the article and approved the submitted version.

## Conflict of interest

The authors declare that the research was conducted in the absence of any commercial or financial relationships that could be construed as a potential conflict of interest.

## Publisher’s note

All claims expressed in this article are solely those of the authors and do not necessarily represent those of their affiliated organizations, or those of the publisher, the editors and the reviewers. Any product that may be evaluated in this article, or claim that may be made by its manufacturer, is not guaranteed or endorsed by the publisher.
